# Supplementary material for: Generating rhythm game music with jukebox
Source: Front Artif Intell. 2024 Jul 5;7:1296034. doi: 10.3389/frai.2024.1296034 (PMC11258020; doi:10.3389/frai.2024.1296034)
Supplement: Supplementary file 2 [file Data_Sheet_1.docx]

Supplementary Material

Generating Rhythm Game Music with Jukebox

Nicholas Yan*

*** Correspondence:** Nicholas Yan: cubeymath@gmail.com

# Jukebox Parameters

The link for the Google Colab notebook used to generate excerpts is as follows:

https://colab.research.google.com/drive/1sJda9v46gNzBc7m59MP5zn63AWc-axCY?usp=sharing.

In the “input” column, parameters that were left blank were dependent on the specific song used. These columns only affect the file’s location within the Google Drive, not the song itself.

**Table S1**

*Overview of Jukebox parameters and example inputs*

| **Name** | **Function** | **Input** |
| --- | --- | --- |
| model | Which model Jukebox uses; 5b is the newest available version that does not support lyric generation | 5b |
| hps.n_samples | How many audio files outputted | 1 |
| hps.name | Name of output folder |  |
| speed_upsampling | Sacrifices quality for speed while sampling | Yes |
| mode | “Ancestral” creates from scratch, “primed” uses your audio file as input | primed |
| audio_file | If “primed” selected, this is the audio file name (must list file path) |  |
| prompt_length_in_seconds | Length of initial audio file before Jukebox begins producing | 30 |
| sample_length_in_seconds | Length of full sample | 90 |
| select_artist and select_genre | Sample influenced by an artist/a genre’s style | *(left blank)* |
| sampling_temperature | Value between 0 and 1 that controls the randomness/creativity of output; suggested between 0.96 and 0.999 | 0.98 |
| disconnect_runtime_after_finish | Automatically disconnects from Jukebox after samples finish generating | Yes |

# Song List

**Table S2**

*List of artcore songs used with basic information*

| **#** | **Name** | **Key** | **Tempo (BPM)** | **Time Signature** |
| --- | --- | --- | --- | --- |
| 1 | xi- Anima | F minor | 183.5 | 4/4 |
| 2 | cosMo@暴走P - Ωκεανος  *(read: Oceanus)* | G# minor | 196 | 4/4 |
| 3 | M2U & NICODE- Lune | Eb minor | 160 | 4/4 |
| 4 | Ice vs. Morimori Atsushi - RE:UNION -Duo Blade Against- | B minor | 202 | 4/4 |
| 5 | Ice- Amber Wishes | C minor | 196 | 4/4 |
| 6 | NeLIME- Leviathan | A minor | 180 | 4/4 |
| 7 | Alex Vourtsanis- Sunset | Bb minor | 180 | 4/4 |
| 8 | Ice- Parodia Sonatina Var.II | C# minor | 170 | 4/4 |

**Table S3**

*List of orchestral songs used with basic information*

| **#** | **Name** | **Key** | **Tempo (BPM)** | **Time Signature** |
| --- | --- | --- | --- | --- |
| 9 | Tzu-Chieh Wen- 桜色の夢  *(read: Sakura iro no yume)* | Bb minor | 70 | 4/4 |
| 10 | onoken ft. Rin- Fluquor | G minor | 125 | 3/4 |
| 11 | himmel - Carnation | A minor | 118 | 3/4 |
| 12 | V.K.- Reverse-Parallel Universe | F# minor | 120 | 4/4 |
| 13 | Kitkit Lu- Graduation Song | F minor | 122 | 3/4 |
| 14 | Sta- Platinum | C# minor | 100 | 4/4 |
| 15 | V.K.- Evolution Era | F# minor | 90 | 4/4 |
| 16 | Edmud Fu- Living in the One | F minor | 68 | 4/4 |

**Table S4**

*List of all songs. The arrangements listed below were used as input for Jukebox.*

| **#** | **Name** | **Arranger + Link** |
| --- | --- | --- |
| 1 | xi- Anima | Ayato Fujiwara  https://www.youtube.com/watch?v=HrKBE73aWHc |
| 2 | cosMo@暴走P - Ωκεανος  *(read: Oceanus)* | Ayato Fujiwara  https://www.youtube.com/watch?v=-yLQ25RuBvQ |
| 3 | M2U & NICODE- Lune | phyxinon  https://www.youtube.com/watch?v=wlsBbIoPjKw |
| 4 | Ice vs. Morimori Atsushi- RE:UNION -Duo Blade Against- | ALFetite (original: phyxinon)  https://www.youtube.com/watch?v=JtlqUgx28tI |
| 5 | Ice- Amber Wishes | Hakubun  https://www.youtube.com/watch?v=-x0tX9qGMQk |
| 6 | NeLIME- Leviathan | ALFetite (original: phyxinon)  https://www.youtube.com/watch?v=x555-7N3H3A |
| 7 | Alex Vourtsanis- Sunset | ALFetite (original: phyxinon)  https://www.youtube.com/watch?v=_syC8rTT6aQ |
| 8 | Ice- Parodia Sonatina Var.II | Luca  https://www.youtube.com/watch?v=NxYmtw2GpgY |
| 9 | Tzu-Chieh Wen- 桜色の夢  *(read: Sakura iro no yume)* | Ayato Fujiwara  https://www.youtube.com/watch?v=_dfLdIsRNdM |
| 10 | onoken ft. Rin- Fluquor | Ayato Fujiwara  https://www.youtube.com/watch?v=hXR2WBSDRPM |
| 11 | himmel - Carnation | K# Piano  https://www.youtube.com/watch?v=38rBM4vr3yM |
| 12 | V.K.- Reverse-Parallel Universe | Ayato Fujiwara  https://www.youtube.com/watch?v=GliDmG1h5gI |
| 13 | Kitkit Lu- Graduation Song | ぴおーね  https://www.youtube.com/watch?v=1HXSLPrku8Y |
| 14 | Sta- Platinum | Salamanz  https://www.youtube.com/watch?v=JZYcrVYK8TM |
| 15 | V.K.- Evolution Era | Salamanz  https://www.youtube.com/watch?v=LTf1f6k8JEE |
| 16 | Edmud Fu- Living in the One | Salamanz  https://www.youtube.com/watch?v=shL3TZJYvWw |

# Survey Questions

The following hyperlink leads to the survey in an electronic format. Note that this is a copy, and no responses have been saved:

https://docs.google.com/forms/d/e/1FAIpQLSc9wT-vWcRjEROTI2WGz1FTqHrAiky9TF3jUMtASjnYV4KNkA/viewform

Page 1

The purpose of this survey is to compare people’s perceptions of music generated by humans and AI. You will not receive any immediate benefits from taking this survey. However, your responses may be used in a research study which will expand the recorded repertoire of music that neural networks can create. Participation is voluntary, and no identifiable information will be collected nor stored.

You are recommended to complete this survey in one sitting in a quiet place where you can clearly hear the audio. This survey should take no longer than 15 minutes to complete.

If you have any questions, feel free to contact me (████) at ████@████.org or my AP Research teacher (█████) at █████@█████.org.

By clicking ‘next,’ you give consent for your responses to be included within the research paper I will write. **You also indicate that you are over the age of 18 or have parental consent to complete the survey.**

Page 2

Listen to the following audio files below and answer the questions. There are a total of sixteen audio recordings on this page. To reduce bias, you have not been told who created each recording (human or AI).

For each ranked choice question, 1 is the worst and 5 is the best. For each free response question, feel free to write as little or as much as you like. Citing timestamps (e.g. 0:06) would be especially helpful if possible.

Each of the 16 questions follows the format below:

[Audio file; hyperlink]

1. Was the creator of the recording a human or AI? *(required)*

     A. Human

     B. AI

2. Rate the musicality of the recording. A higher number means more musicality. *(required)*

1 2 3 4 5

3. What determined your response to the previous question?

(free response)

Page 3

Do you have any additional comments?

(free response)
